# Unexpected infectious complication following AVM embolization: *E. coli* brain abscess

**DOI:** 10.1016/j.ensci.2025.100594

**Published:** 2025-11-13

**Authors:** Andrii Netliukh, Andrian Sukhanov, Nana Tchantchaleishvili

**Affiliations:** a BAU Batumi International University, Batumi, Georgia; b 1st Lviv Territorial Medical Union, Lviv, Ukraine; c Danylo Halytskyi Lviv National Medical University, Lviv, Ukraine; d Grenoble Institute of Neurosciences, Grenoble, France

**Keywords:** Arteriovenous malformation, Brain abscess, Endovascular embolization, *E. coli*

## Abstract

**Background:**

Brain arteriovenous malformations (AVMs) are rare vascular anomalies managed with surgery, radiosurgery, or endovascular embolization. Post-embolization intracranial infections are extremely uncommon, especially *Escherichia coli* abscesses in immunocompetent adults.

**Case presentation:**

We report a 37-year-old man with a giant left frontal AVM treated with staged Onyx® embolization. One month after the final session, he developed a left frontal brain abscess with motor aphasia and right-sided hemiparesis. Cultures grew hemolytic *E. coli* sensitive to multiple antibiotics. Recurrence after initial drainage necessitated complete AVM and nidus resection, followed by prolonged targeted antibiotic therapy, leading to resolution and neurological recovery.

**Literature review:**

Intracranial *E. coli* infections and post-AVM embolization abscesses are rare, typically associated with systemic or local immunosuppression. Our case is among the first describing hemolytic *E. coli* abscess after Onyx® embolization in a healthy adult.

**Conclusion:**

Retained embolic material, local inflammation, and blood–brain barrier disruption may facilitate infection. Early recognition and total removal, and prolonged targeted antibiotics are crucial to prevent recurrence and ensure favorable outcomes.

## Highlights

• This review consolidates all current evidence on concurrent trigeminal and facial nerve compression.
• Analysis of published series reveals consistent patterns of dual neurovascular conflict and anatomical variation.
• Comparison of imaging and operative findings highlights persistent diagnostic limitations and pitfalls.
• The review clarifies evidence-based surgical principles to optimize microvascular decompression strategy.
• Findings provide clinicians with practical guidance for diagnosing and managing complex dual compression syndromes.

## 1 Introduction

Brain arteriovenous malformations (AVMs) are rare congenital vascular anomalies characterized by direct arteriovenous shunting through a nidus, bypassing the capillary bed and creating high-flow, low-resistance circuits. They account for approximately 1–2 % of all intracranial vascular lesions and represent a leading cause of spontaneous intracerebral hemorrhage in young adults [1,2]. The annual risk of rupture ranges from 1 % to 4 %, but may rise significantly in lesions with unfavorable angioarchitectural features such as deep location, exclusive deep venous drainage, venous stenosis, or associated aneurysms—parameters incorporated into grading systems like the Spetzler–Martin and Buffalo scales [[3], [4], [5]].

Management of AVMs remains complex and individualized. Treatment options include microsurgical resection, stereotactic radiosurgery, and endovascular embolization, often used in combination depending on the lesion's anatomy and risk profile [6,7]. Endovascular embolization plays a pivotal role, serving as a curative, adjunctive, or palliative strategy for nidus reduction or high-risk feeder occlusion [8]. Among available embolic agents, ethylene-vinyl alcohol copolymer (Onyx®) is favored for its controlled injection, non-adhesive nature, and radiopacity [9]. Nonetheless, it carries potential risks such as vessel perforation, non-target embolization, hemorrhage, catheter retention, and thromboembolic events [10,11].

Infectious complications following AVM embolization are exceedingly rare. When they occur, they are typically superficial, related to vascular access or soft-tissue contamination, while intracranial infections are estimated to occur in less than 0.1 % of neurointerventional procedures [12]. Most reported intracranial infections involve Gram-positive skin flora such as *Staphylococcus aureus* or *Streptococcus* species [13,14]. Gram-negative pathogens are exceptional; *Escherichia coli* brain abscesses are typically confined to neonates, elderly, or immunocompromised patients, usually secondary to systemic infection originating from urinary or gastrointestinal sources [[15], [16], [17]].

In adults without systemic infection, *E. coli* brain abscesses are exceedingly uncommon. The potential mechanism in the post-embolization setting may involve repeated catheterization, disruption of the blood–brain barrier, or bacterial colonization of retained synthetic material such as Onyx®, which may act as a substrate for biofilm formation [18,19].

Here, we describe a unique case of a 37-year-old immunocompetent man who developed a recurrent *E. coli* brain abscess following staged Onyx® embolization of a giant left frontal AVM. To our knowledge, this represents one of the first reported cases of a hemolytic *E. coli* brain abscess associated with embolized AVM tissue. This case underscores the importance of vigilance for delayed infectious complications, highlights the potential role of embolic material in sustaining infection, and illustrates the need for multidisciplinary management when conservative therapy fails.

To contextualize this case, two structured literature searches were performed. The first search aimed to identify all reported intracranial E. Coli infections in adults, regardless of etiology. The second search focused specifically on brain abscess formation following arteriovenous malformation (AVM) embolization with any embolic agent. Searches were conducted in PubMed, Scopus, and Google Scholar databases up to June 2025 using the following combined terms and Boolean operators: (“Escherichia coli” AND (“brain abscess” OR “intracranial infection”)) OR (“AVM embolization” AND (“Onyx” OR “NBCA” OR “coil”) AND (“infection” OR “abscess”)) No language or publication date restrictions were applied. Additional relevant reports were identified through manual screening of reference lists of pertinent articles. For the E. coli dataset (Table 1), inclusion criteria were: (1) confirmed intracranial infection caused by E. coli, (2) adult patients, and (3) available clinical and microbiological details. For the AVM embolization dataset (Table 2), inclusion criteria were: (1) confirmed intracranial abscess following AVM embolization, (2) reported timing of abscess development, and (3) documented pathogen when available. Reports lacking sufficient clinical or microbiological information were excluded. Both searches were performed independently and synthesized descriptively.

**Table 1:** Blood test parameters during the patient's treatment course.

Parameter	03.07.2023	10.07.2023	12.09.2023	02.10.2023	04.12.2023	20.12.2023
Hemoglobin (g/L)	136	106	135	131	137	121
Hematocrit	0.41	0.31	0.41	0.40	0.40	0.37
Erythrocytes (×10^12^/L)	4.68	3.72	4.65	4.76	4.84	4.48
Platelets (×10^9^/L)	373	229	339	384	295	192
Leukocytes (×10^9^/L)	16.65	9.41	18.47	7.51	13.94	17.41
Bilirubin (μmol/L)	3.55	18.5	5.9	6.95	10.0	5.07
Aspartate Aminotransferase (U/L)	19.4	29.3	28.8	25.1	17.3	33.3
Alanine Aminotransferase (U/L)	64.0	40.1	34.2	19.0	14.3	45.4
Creatinine (μmol/L)	65.1	77.0	51.5	64.0	70.5	55.83
Urea (mmol/L)	4.4	4.7	3.12	2.2	3.19	6.37
C-reactive Protein (mg/mL)	59.8	31.6	29.3	11.75	118	4.0
Procalcitonin (ng/mL)	<0.10	<0.10	<0.10	<0.10	<0.10	<0.10

**Table 2:** Results of culture of abscess contents: antibiotic susceptibility of hemolytic *Escherichia coli* (10^8^ CFU/mL).

Antibiotic Susceptibility	
Beta-lactams	
Ampicillin	Sensitive (S)
Amoxicillin-Clavulanate	Sensitive (S)
Ticarcillin-Clavulanate	Sensitive, Increased Exposure (IE)
Cefotaxime	Sensitive (S)
Ceftriaxone	Sensitive (S)
Ceftazidime	Sensitive (S)
Cefepime	Sensitive (S)
Imipenem	Sensitive (S)
Meropenem	Sensitive (S)

Aminoglycosides	
Amikacin	Sensitive (S)
Gentamicin	Sensitive (S)

Fluoroquinolones	
Ciprofloxacin	MIC provided
Levofloxacin	Sensitive (S)

Others	
Trimethoprim-Sulfamethoxazole	Resistant (R)

**S** – Sensitive; **R** – Resistant; **IE** – Sensitive, Increased Exposure; **MIC** – Minimum Inhibitory Concentration provided instead of categorical result.

### 1.1 Case presentation

A 37-year-old right-handed man with no significant past medical history initially presented in March 2022 with new-onset generalized seizures, progressive memory impairment, episodic headaches, and generalized weakness. The neurological examination revealed mild cognitive deficits, with a Glasgow Coma Scale (GCS) score of 15/15.

**Fig. 1:**
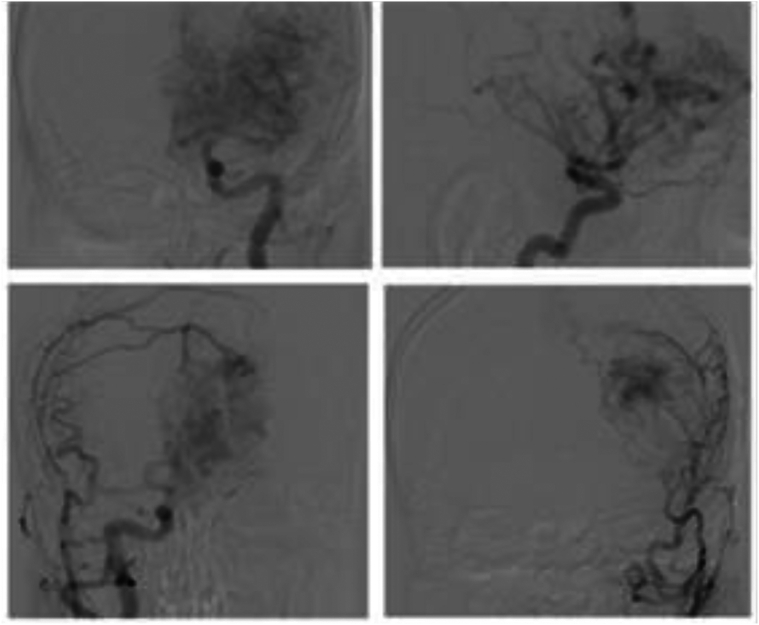
Left frontal lobe arteriovenous malformation (AVM) measuring 63 × 51 mm, classified as Spetzler-Martin grade V with a Buffalo score of 5. Supplied by the left and right internal carotid arteries (ICA), common carotid arteries (CCA), and vertebral arteries (VAs). Venous drainage occurs through superficial and deep veins into the sagittal and transverse sinuses, with a deep vein aneurysm measuring 13.1 × 13.6 mm.

Brain MRI showed a giant AVM in the left frontal lobe, measuring 63 × 51 mm, classified as Spetzler–Martin grade V and Buffalo grade 5. The AVM was supplied by bilateral internal and common carotid arteries and the vertebral arteries. Venous drainage was through both superficial and deep systems into the sagittal and transverse sinuses. A 13.1 × 13.6 mm deep venous aneurysm was also identified (Fig. 1). Due to the AVM's size and complexity, staged endovascular embolization with Onyx® was decided.

**Fig. 2:**
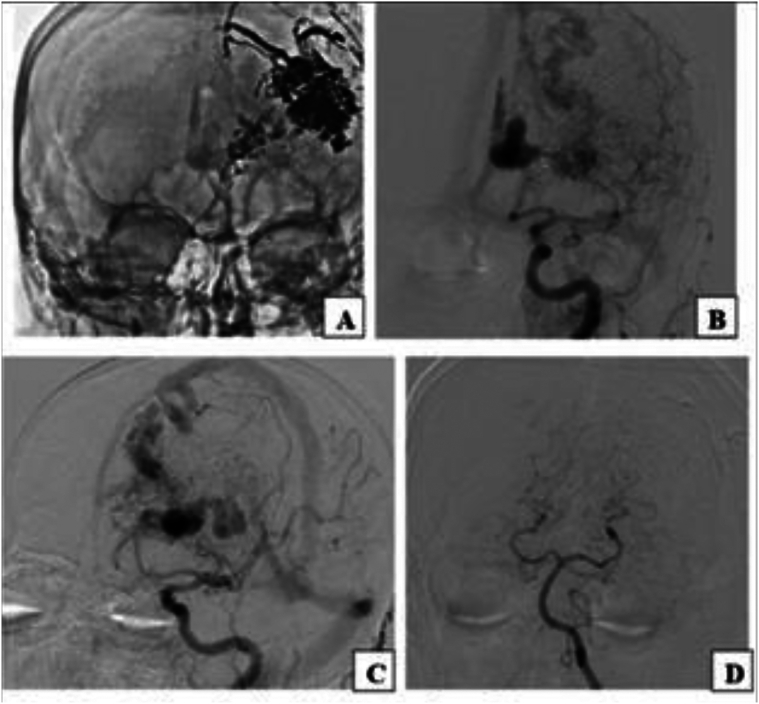
Partially embolized AVM. **A.** Frontal direct projection (without subtraction); **B.** Frontal direct projection (basin of the left ICA); **C.** Left oblique projection (basin of the left ICA); **D.** Frontal direct projection (vertebrobasilar basin).

The patient underwent three-stage embolization in September 2022, December 2022, and April 2023. All procedures were technically successful and well tolerated. Post-embolization imaging is shown in Fig. 2.

**Fig. 3:**
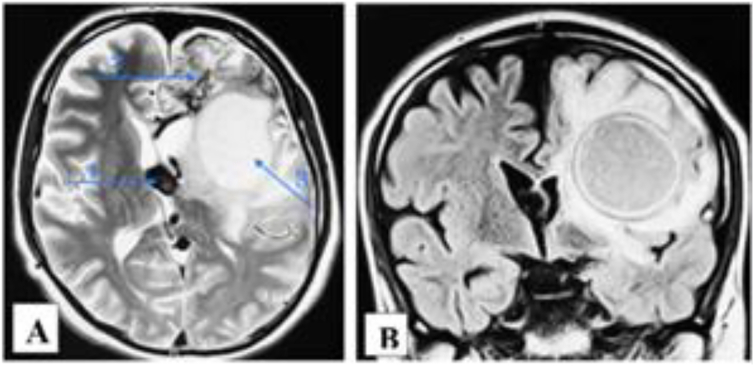
AVM-associated changes and complications. **A.** Axial plane showing midline displacement up to 14 mm to the right, deep vein aneurysm 13.2 × 13.6 mm (1), embolized part of the AVM (2), and abscess measuring 53 × 40 × 30 mm (3). **B.** Coronal plane depiction of the same findings.

**Fig. 4:**
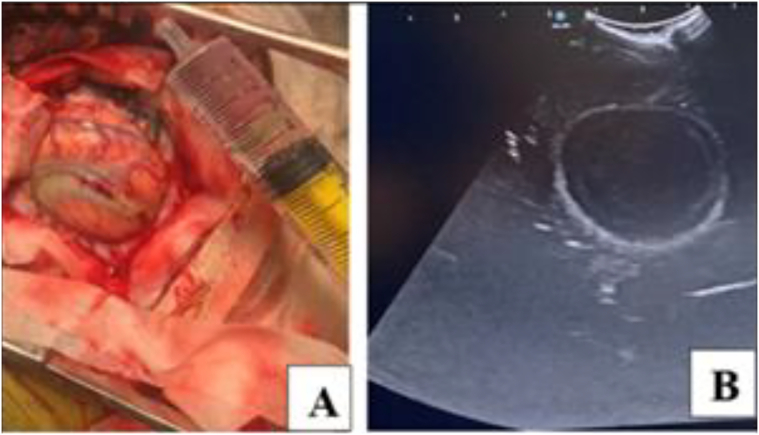
Intraoperative imaging. **A.** Intraoperative photograph; **B.** Ultrasound image.

In June 2023, approximately one month after the final embolization, he was readmitted with fever (39 °C), motor aphasia, right facial asymmetry, and right-sided hemiparesis. Brain MRI revealed a 53 × 40 × 30 mm abscess in the left frontal lobe surrounding the embolized nidus, associated with a 14 mm midline shift (Fig. 3). Despite empiric antibiotic therapy and interventions to control intracranial pressure, the patient's neurological condition progressively worsened, ultimately necessitating surgical intervention. Throughout hospitalization, serial laboratory investigations revealed dynamic changes in inflammatory markers, liver enzymes, and hematologic parameters, closely correlating with the evolving stages of infection and therapeutic response (Table 3). Surgical management involved a left frontal craniotomy, performed under intraoperative ultrasound guidance to navigate and preserve the AVM-associated vasculature. Approximately 40 mL of purulent material was aspirated, a sample was sent for culture, and a continuous inflow–outflow drainage system was placed (Fig. 4). Cultures grew hemolytic *Escherichia coli* (10^8^ CFU), sensitive to ceftriaxone, meropenem, ciprofloxacin, and amikacin (Table 4). Blood and urine cultures, transthoracic echocardiography, full-body imaging, and oral and ENT evaluations were all unremarkable. The patient reported no gastrointestinal or urinary symptoms. Lumbar puncture was not performed due to the patient's refusal.

**Table 3:** Reported cases of intracranial infections with *Escherichia coli* [[24], [25], [26], [27], [28], [29], [30], [31], [32], [33]].

Authors	Age (yrs)	Gender	Immunosuppression	Underlying Condition	Potential Source	Lesion	Treatment	Outcome
Bakker et al. (1995)	88	F	None	Anal fissure	UTI	Subdural empyema	Drainage	Died
Hirano et al. (1995)	86	M	None	Chronic cholecystitis	Cholecystitis	Subdural empyema	Drainage	Died
Rickert et al. (2000)	52	M	Steroids	Dilated cardiomyopathy, diabetes	None	Cerebral abscess	Craniotomy	Died
Nishi et al. (2005)	76	M	None	Polycystic kidneys	Renal cyst infection	Subdural empyema	Drainage	Alive
Bachmeyer et al. (2005)	55	M	None	Esophageal cancer	None	Subdural empyema	–	Died
Doepp et al. (2006)	67	M	None	Patent foramen ovale	Perianal abscess	Cerebral abscess	–	Alive
Adamides et al. (2007)	91	M	None	Chronic SDH, diabetes	Neurosurgery	Subdural empyema	Drainage	Died
Narita et al. (2009)	80	M	None	Splenectomy/gastrectomy	UTI	Subdural empyema	Drainage	Alive
Redhu et al. (2011)	48	M	None	None	None	Subdural empyema	Craniotomy	Died
Shah et al. (2019)	53	M	None	Cirrhosis, AVM rupture	AVM embolization	Cerebral abscess	Removal	Alive

**Table 4:** Reported cases of brain infections following AVM embolization [[33], [34], [35], [36], [37], [38], [39], [40]].

Authors	Age (yrs)	Gender	AVM Location	Hemorrhage	Embolization Sessions	Time to Abscess	Pathogen
Mourier et al. (1993)	24	F	Right frontal lobe	Yes	2 (NBCA)	4 months	*S. aureus*
Pendarkar et al. (2006)	30	M	Right frontoparietal	Yes	4 (NBCA)	6 months	*P. aeruginosa*
Chagla et al. (2008)	24	M	Left parietal	No	1 (NBCA)	4 years	Unknown
Sharma et al. (2011)	38	M	Right parieto-occipital	Yes	1 (NBCA)	10 months	Unknown
Sharma et al. (2011)	25	F	Left frontal	No	1 (NBCA)	5 months	B. cepacian
Khoshnevisan et al. (2014)	21	F	Left parietal	No	1 (NBCA)	5 months	*E. coli*
Jabre et al. (2019)	68	M	Left occipital	No	Coils/NBCA (1)	10 months	*E. faecalis*
Shah et al. (2020)	53	M	Left temporo-occipital	Yes	Onyx (2)	5 weeks	*E. coli*

NBCA = N-butyl cyanoacrylate.

**Fig. 5:**
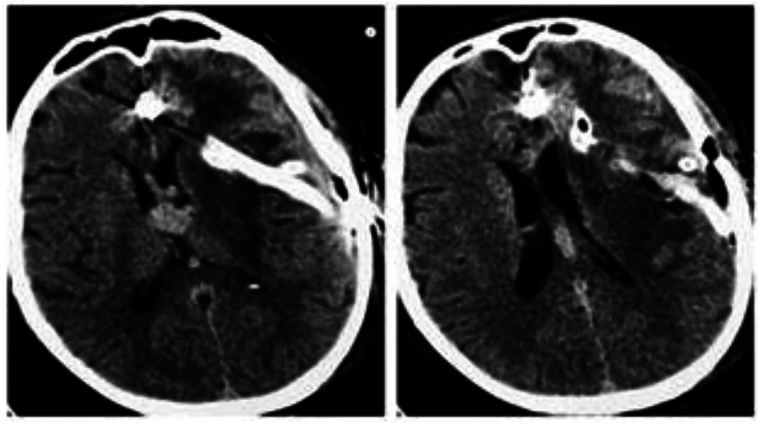
Postoperative changes in the left frontal lobe, showing encephalomalacia with cystic changes and small ischemic foci. There is a noticeable mass effect in the anterior frontal regions compared to the previous examination, resulting in the displacement of midline structures up to 6 mm.

Postoperatively, the patient's motor aphasia and hemiparesis gradually improved. Follow-up MRI showed a reduction in abscess size and improvement of the midline shift to 6 mm (Fig. 5). He completed four weeks of intravenous ceftriaxone followed by oral antibiotics.

**Fig. 6:**
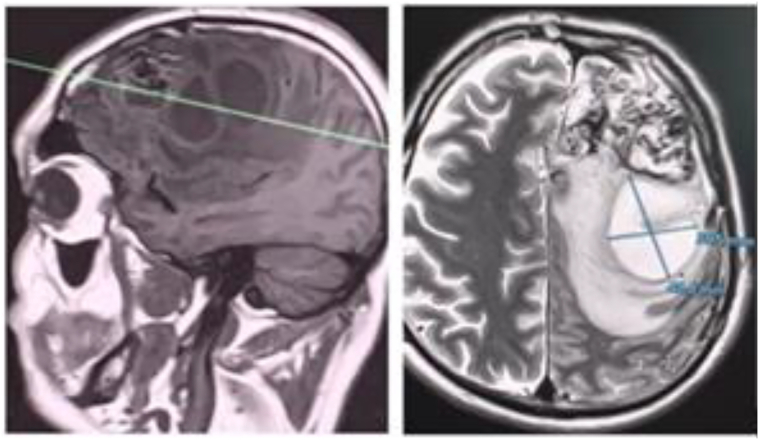
Partially embolized left frontal AVM with a left frontal lobe cyst, likely representing an abscess, measuring 54 × 47 mm. Midline structures are displaced up to 10 mm.

**Fig. 7:**
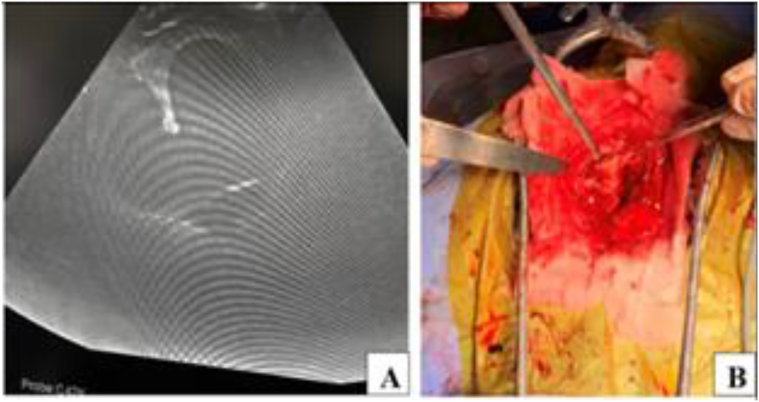
Intraoperative imaging and procedure. **A.** Ultrasound image of the lesion; **B.** Intraoperative photograph showing abscess removal.

In September 2023, the patient was readmitted with recurrent fever and new right-sided weakness. Repeat MRI demonstrated recurrence of the abscess (54 × 47 mm) despite ongoing prophylactic antibiotics (Fig. 6). A second left frontal craniotomy was performed, allowing complete excision of the abscess capsule (∼50 mL), resection of the AVM, including embolized segments, and placement of a new drainage catheter (Fig. 7). Cultures again revealed a hemolytic strain of *E. coli* (10^4^ CFU) with identical sensitivities. Repeat systemic and local infectious evaluations were again negative.

**Fig. 8:**
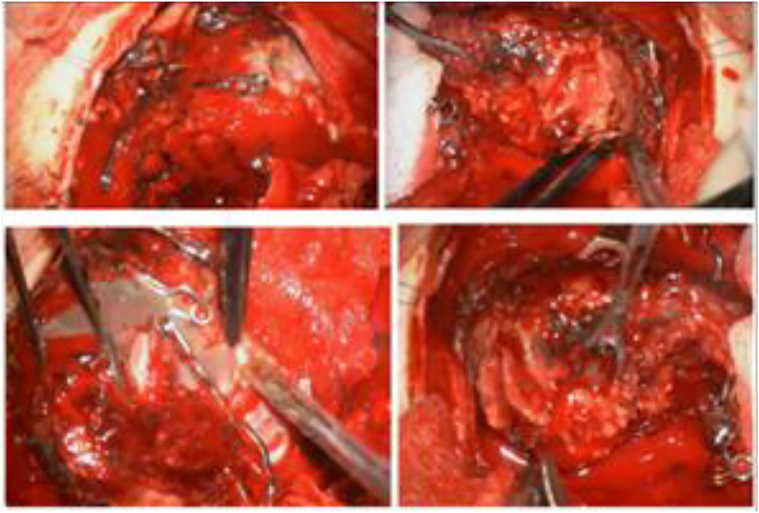
Surgical excision of the partially embolized malformation with removal of infected contents.

The patient completed a four-week course of intravenous amoxicillin–clavulanate, followed by oral therapy. CT imaging revealed cystic encephalomalacia in the left frontal lobe with a minimal 2 mm residual midline shift and no evidence of residual abscess (Fig. 8). His neurological status continued to improve, with only mild residual motor aphasia and right arm monoparesis. Follow-up digital subtraction angiography in June 2024 showed complete obliteration of the AVM. As of his most recent follow-up in June 2025, the patient remained stable, seizure-free on antiseizure therapy, and with no signs of infectious recurrence.

Table 5 summarizes the chronological sequence of the patient's clinical course, including the diagnosis of AVM, staged embolization procedures, onset of infection, surgical interventions, and follow-up outcomes.

**Table 5:** Timeline of major clinical events, treatments, and outcomes.

Date / Period	Event	Key Details
March 2022	Initial presentation	New-onset seizures, cognitive impairment → MRI: large left frontal AVM
Sept 2022 – Apr 2023	Three-stage Onyx ® embolization	Technically successful, no early complications
June 2023	First hospitalization	Fever, right hemiparesis → MRI: abscess 53 × 40 × 30 mm
July 2023	First craniotomy	Drainage of abscess; Onyx material retained; *E. coli* isolated → antibiotics started
Sept 2023	Recurrence	MRI: recurrent abscess 54 × 47 mm despite antibiotics
Oct 2023	Second craniotomy	Complete excision of AVM nidus and abscess capsule
Dec 2023 – June 2024	Recovery and follow-up	MRI: no abscess; DSA: complete AVM obliteration
June 2025	Long-term outcome	Stable, seizure-free, mild motor aphasia

## 2 Discussion

Brain arteriovenous malformations (AVMs) are high-flow vascular anomalies that carry a significant risk of hemorrhage, particularly in large and high-grade lesions. Endovascular embolization is widely used in the management of AVMs, either as a standalone therapy or adjunct to surgical resection or radiosurgery, especially for complex or high-risk cases [20]. Although generally safe, endovascular interventions carry potential complications, including hemorrhage, ischemia, and, less commonly, infection.

Intracranial infection following AVM embolization is extremely rare, with only a few cases reported in the literature [5,6,21]. Most documented infections involve Gram-positive organisms, commonly skin flora such as *Staphylococcus aureus* or *Streptococcus* species, which may be introduced during vascular access or catheter manipulation [22,23]. Our case is unique in identifying hemolytic *Escherichia coli*, a Gram-negative bacterium rarely associated with brain abscesses in immunocompetent adults. A summary of previously reported cases of *E. coli* brain abscesses is presented in Table 3 along with their clinical context, treatment, and outcomes across various etiologies.

The pathogenesis of *E. coli* brain abscess formation in this setting is likely multifactorial. Possible mechanisms include hematogenous spread from transient bacteremia during endovascular procedures, direct contamination of the vascular access system, or secondary infection of necrotic embolized tissue [12,13]. In immunocompetent adults, *E. coli* CNS infection typically requires a predisposing nidus of inflammation or ischemic tissue that facilitates bacterial adhesion and parenchymal invasion [15,16].

Published case reports indicate that *E. coli* abscesses most often occur secondary to urinary tract or gastrointestinal infections, hepatic or biliary sepsis, or endocarditis [[24], [25], [26], [27]]. Nevertheless, several reports describe spontaneous or iatrogenic infections in patients without identifiable systemic sources [[28], [29], [30], [31], [32], [33]]. In our patient, the absence of extracranial infection and the close temporal relationship with Onyx® embolization strongly suggest localized infection of the embolized nidus as the most plausible mechanism. This hypothesis is consistent with previously reported post-embolization abscesses, where retained synthetic embolic materials acted as persistent foreign bodies supporting bacterial colonization and biofilm formation [19,22,23].

In adults, *E. coli* brain abscesses are uncommon and typically occur in the setting of systemic infection, contiguous spread from gastrointestinal or urinary tract sources, or immunosuppression [[24], [25], [26]]. The absence of any such source in our patient, despite thorough systemic and microbiological evaluation, makes this case particularly notable. The patient had no gastrointestinal or urinary symptoms, normal systemic imaging, and unremarkable blood and urine cultures. This strongly suggests that the infection was not community-acquired, but likely developed locally after embolization.

A potential mechanism involves colonization of the embolized nidus itself. Onyx®, the embolic agent used in this case, is a non-biodegradable ethylene-vinyl alcohol copolymer that remains permanently within the cerebral vasculature. While biocompatible, its synthetic surface may promote bacterial adherence and biofilm formation, a well-known process in device-associated infections [19,22]. Biofilms provide bacteria with a protective niche, making them resistant to both host immune responses and antibiotics, which can lead to persistent or recurrent infection despite appropriate therapy [23]. Although Onyx has not commonly been reported as a substrate for biofilm formation in the brain, similar behavior is well documented in prosthetic materials, vascular grafts, and indwelling catheters.

In our case, infection emerged one month after the final embolization session, and despite initial surgical drainage and antibiotic treatment, the abscess recurred three months later. This clinical course highlights the limitations of conservative management when infected embolic material remains in situ. Only after complete resection of the AVM and its embolized nidus, sustained infection control was achieved.

Staged embolization, while safer for large and complex AVMs, may increase the cumulative risk of local inflammation, thrombosis, and procedural contamination. These changes can compromise the blood-brain barrier and create a pro-inflammatory microenvironment, possibly facilitating microbial colonization [9,10]. Furthermore, the use of multiple embolic sessions may increase manipulation of intracranial vessels and lengthen exposure time, raising the theoretical risk of iatrogenic infection, especially in the presence of retained foreign material.

There are no standardized guidelines for managing brain abscesses following AVM embolization, given the rarity of this complication. However, our experience and available literature suggest that early recognition, surgical drainage, prolonged culture-directed antibiotic therapy, and most importantly, resection of the infected nidus are key to successful management. Multidisciplinary collaboration between neurosurgery, interventional neuroradiology, infectious disease, and critical care teams is essential for enhancing outcomes in these complex cases. Complete surgical excision of the infected nidus was ultimately required to achieve infection control.

## 3 Conclusions

Brain abscess following AVM embolization is an uncommon but potentially life-threatening complication that requires high clinical suspicion, especially when neurological deterioration occurs post-procedure. This case highlights that hemolytic *Escherichia coli* can cause brain abscess even in immunocompetent patients without an identifiable systemic infection, suggesting that infection may arise from bacterial colonization of retained non-biodegradable embolic material, such as Onyx. The ability of Onyx® to serve as a persistent nidus for biofilm formation underscores the challenge in eradicating such infections with antibiotics alone.

Effective management necessitates early recognition, aggressive surgical drainage, and prolonged culture-directed antibiotic therapy. Complete surgical excision of the infected AVM nidus and embolic material is often required to achieve lasting infection control and prevent recurrence. Multidisciplinary collaboration and close postoperative monitoring are essential in optimizing outcomes in patients undergoing complex AVM embolization.

## CRediT authorship contribution statement

**Andrii Netliukh:** Writing – review & editing, Writing – original draft, Supervision, Project administration, Data curation. **Andrian Sukhanov:** Writing – review & editing, Writing – original draft, Validation, Resources, Methodology, Formal analysis, Conceptualization. **Nana Tchantchaleishvili:** Writing – review & editing, Writing – original draft, Visualization, Validation, Resources, Project administration, Methodology, Formal analysis.

## Declaration of competing interest

None.

## References

[1] Abecassis I.J., Xu D.S., Batjer H.H. (2014). Natural history of brain arteriovenous malformations: a systematic review. Neurosurg. Focus..

[2] Mohr J.P., Parides M.K., Stapf C. (2014). Medical management with or without interventional therapy for unruptured brain arteriovenous malformations (ARUBA): a multicentre, non-blinded, randomised trial. Lancet.

[3] Spetzler R.F., Martin N.A. (1986). A proposed grading system for arteriovenous malformations. J. Neurosurg..

[4] Soderman M., Holmin S., Andersson T. (2006). Arteriovenous malformations of the brain: a new grading system for endovascular treatment. J. Neurosurg..

[5] Lawton M.T., Rutledge W.C., Kim H. (2015). Brain AVM response to surgery, radiosurgery, and embolization: a review of the literature and meta-analysis. World Neurosurg..

[6] Stapf C., Mast H., Sciacca R.R. (2003). The New York Islands AVM study: design, study progress, and initial results. Stroke.

[7] Hartmann A., Mast H., Mohr J.P. (1998). Determinants of neurological and cognitive status in patients with unruptured brain arteriovenous malformation. Stroke.

[8] van Rooij W.J., Sluzewski M., Beute G.N. (2007). Brain AVM embolization with Onyx. AJNR Am. J. Neuroradiol..

[9] Saatci I., Geyik S., Yavuz K. (2011). Endovascular treatment of brain AVMs with prolonged Onyx injection technique. AJNR Am. J. Neuroradiol..

[10] Abud D.G., Riva R., Nakiri G.S. (2007). Embolization of brain arteriovenous malformations with Onyx: technical aspects. AJNR Am. J. Neuroradiol..

[11] Bervini D., Morgan M.K., Ritson E.A., Heller G.Z. (2016). Surgery for cerebral arteriovenous malformation: effect of supplementary embolisation on surgical performance. J. Clin. Neurosci..

[12] Drazin D., Alexander M.J. (2011). Infection after neuroendovascular procedures. Neurohospitalist.

[13] Sharma B.S., Pandey P., Gupta V. (2001). Intracranial abscess after embolization of brain arteriovenous malformation: case report. Neurosurgery.

[14] Lange N., Berndt M., Jörger A.K. (2020). Incidence and mortality of brain abscesses in Denmark. Clin. Microbiol. Infect..

[15] Kim K.S. (2003). Pathogenesis of bacterial meningitis: from bacteraemia to neuronal injury. Nat. Rev. Neurosci..

[16] Bichon A., Aubry C., Dubourg G. (2018). *Escherichia coli* spontaneous community-acquired meningitis in adults. Int. J. Infect. Dis..

[17] van Samkar A., Brouwer M.C., van der Ende A. (2015). *Escherichia coli* meningitis in adults: clinical features and outcome. J. Inf. Secur..

[18] Raper D.M., Starke R.M., Komotar R.J. (2013). Surgical management of cerebral arteriovenous malformations. Nat. Rev. Neurol..

[19] Hall-Stoodley L., Costerton J.W., Stoodley P. (2004). Bacterial biofilms: from the natural environment to infectious diseases. Nat. Rev. Microbiol..

[20] van Rooij W.J., Sluzewski M. (2010). Curative embolization of brain AVMs with Onyx: patient selection, technique, and results. AJNR Am. J. Neuroradiol..

[21] Brouwer M.C., van de Beek D. (2017). Epidemiology, diagnosis, and treatment of brain abscesses. Curr. Opin. Infect. Dis..

[22] Darouiche R.O. (2004). Treatment of infections associated with surgical implants. N. Engl. J. Med..

[23] Matar W.Y., Jafari S.M., Restrepo C. (2010). Diagnosis, management, and prevention of prosthetic joint infections: a review. Surg. Technol. Int..

[24] Bakker D.J., Tulleken C.A., Groen R.J.M. (1995). Subdural empyema due to *E. Coli* in an elderly woman. J. Inf. Secur..

[25] Hirano Y., Horiguchi T., Uchida Y. (1995). *E. Coli* subdural empyema secondary to cholecystitis. Neurol. Med. Chir. (Tokyo).

[26] Rickert C.H., Kopp H.G., Jellinger K. (2000). Fatal cerebral abscess caused by *E. Coli* in an immunocompromised patient. Clin. Neuropathol..

[27] Nishi K., Tanaka Y., Takasaki K. (2005). Subdural empyema caused by renal cyst infection with *E. coli*. J. Clin. Neurosci..

[28] Bachmeyer C., Daviot B., Wendum D. (2005). Fatal *E. coli* subdural empyema in an immunocompetent adult. Scand. J. Infect. Dis..

[29] Doepp F. (2006). Brain abscess caused by *E. coli* in an adult with patent foramen ovale. Clin. Neurol. Neurosurg..

[30] Adamides A.A. (2007). Subdural empyema following neurosurgery in an elderly diabetic man. Surg. Neurol..

[31] Narita N. (2009). Subdural empyema in a postsplenectomy patient with *E. coli* UTI. Intern. Med..

[32] Redhu N.S. (2011). Spontaneous subdural empyema in a healthy adult: case report. Indian J. Neurotrauma.

[33] Shah A. (2019). Brain abscess in a cirrhotic patient following AVM rupture and embolization. Neurosurg. Rev..

[34] Mourier K.L. (1993). Brain abscess after embolization of cerebral AVM. J. Neurosurg..

[35] Pendarkar A.V. (2006). Post-embolization abscess formation in a treated AVM. Interv. Neuroradiol..

[36] Chagla A. (2008). Brain abscess formation years after AVM embolization: a rare event. Br. J. Neurosurg..

[37] Sharma B.S. (2011). Cerebral abscess following AVM embolization: two case reports. Neurosurgery.

[38] Khoshnevisan A. (2014). Brain abscess due to *E. coli* after AVM embolization. Interv. Neuroradiol..

[39] Jabre A. (2019). Late brain abscess after AVM embolization with NBCA and coils. Surg. Neurol. Int..

[40] Shah A. (2020). Brain abscess after Onyx embolization of a cerebral AVM. Neurosurg. Rev..

